# Prolonged T-Piece Spontaneous Breathing Trial and Extubation Outcomes in Patients Received Prolonged Mechanical Ventilation

**DOI:** 10.3390/medicina61030412

**Published:** 2025-02-26

**Authors:** Tsung-Ming Yang, Yu-Hung Fang, Chia-Hao Chang, Shih-Jiun Lin, Cheng-Chi Liu, David Ming Then Tsai, Chun-Liang Lin, Chieh-Mo Lin, Yung-Chien Hsu

**Affiliations:** 1 Division of Pulmonary and Critical Care Medicine, Chang Gung Memorial Hospital, Chiayi 613016, Taiwan; n120633@cgmh.org.tw (T.-M.Y.); 8902062@cgmh.org.tw (Y.-H.F.); 2 School of Traditional Chinese Medicine, College of Medicine, Chang Gung University, Taoyuan 333423, Taiwan; linchunliang@cgmh.org.tw; 3 Department of Respiratory Care, Chang Gung University of Science and Technology, Chiayi 613016, Taiwan; 4 Department of Nursing, Chang Gung University of Science and Technology, Chiayi 613016, Taiwan; chchang@mail.cgust.edu.tw; 5 Department of Nephrology, Chang Gung Memorial Hospital, Chiayi Branch, Chiayi 613016, Taiwan; jiun0718@gmail.com (S.-J.L.); gnoo0102@gmail.com (C.-C.L.); d10705@hotmail.com (D.M.T.T.); 6 Kidney and Diabetic Complications Research Team (KDCRT), Chang Gung Memorial Hospital, Chiayi 613016, Taiwan; 7 Center for Shockwave Medicine and Tissue Engineering, Chang Gung Memorial Hospital, Kaohsiung 833253, Taiwan; 8 College of Medicine, Chang Gung University, Taoyuan 333423, Taiwan

**Keywords:** spontaneous breathing trial, extubation failure, prolonged mechanical ventilation

## Abstract

*Background and Objectives:* Unassisted breathing through a T-piece was the most used spontaneous breathing trial (SBT) in endotracheal intubated prolonged mechanical ventilation (PMV) patients. However, the optimal duration of an SBT in PMV patients remains uncertain. In this study, we compared the extubation outcome between a 12 h T-piece SBT and a 24 h T-piece SBT in PMV patients. *Materials and Methods:* We reviewed the medical records of PMV patients who were extubated after passing a 12 h or 24 h T-piece SBT. The extubation, weaning, and hospital outcomes between the 12 h T-piece SBT group and the 24 h T-piece SBT group were compared. Kaplan–Meier survival plots and Cox proportional hazard models were used to evaluate the risk of extubation failure between groups. *Results:* In this study, 120 patients were extubated after passing the 12 h T-piece SBT and 234 patients were extubated after passing 24 h T-piece SBT. Patients in the 24 h T-piece SBT group had higher APACHE II score and lower Glasgow coma scale upon RCC arrival than patients in the 12 h T-piece SBT group. There was no difference in gender, age, or ventilator days before extubation between these two groups of patients. After extubation, patients in the 12 h T-piece SBT group and 24 h T-piece SBT group had similar extubation failure rates within 5 days (26.7% vs. 26.1%, *p* = 0.904). There was no difference in the RCC weaning rate (85% vs. 85.5%, *p* = 0.929) and hospital mortality rate (19.8% vs. 21.8%, *p* = 0.821) between the 12 h T-piece SBT group and the 24 h T-piece SBT group. Subgroup analysis showed that 24 h T-piece SBT was associated with a lower extubation failure rate in PMV patients with myocardial infarction or heart failure, but not in older PMV patients or those with cerebrovascular disease. *Conclusions:* The extubation and weaning outcomes were similar in PMV patients extubated after passing 12 h T-piece SBT or 24 h T-piece SBT.

## 1. Introduction

Progress in critical care has improved the survival outcomes of critically ill patients. However, after the critical illness has been stabilized, a substantial proportion of these patients were either partially or completely dependent on mechanical ventilation. Prolonged mechanical ventilation (PMV) was commonly defined as at least 6 h per day of mechanical ventilation for more than 21 consecutive days [1,2,3]. The prevalence of PMV in all mechanically ventilated patients is around 5–14%, depending on the study region and the health insurance reimbursement policies [4,5,6,7]. Patients who received PMV have a higher hospital mortality rate than patients who did not receive PMV [7]. Even after they were discharged from the hospital, patients who received PMV had higher medical resource utilization, total health care costs, and post-discharge mortality rates than those who received mechanical ventilation for a shorter period [7,8]. This relatively higher prevalence and higher medical resource utilization in PMV patients resulted in significant healthcare and economic burdens.

The Respiratory Care Center (RCC) is a hospital-based specialized weaning center for PMV patients whose medical conditions were stabilized and ready for weaning attempts [9]. The weaning process for PMV patients included a gradual reduction of ventilator support, followed by a daily spontaneous breathing trial (SBT). Tracheostomy is thought to provide advantages over endotracheal intubation and has been increasingly used [10]. However, a substantial proportion of PMV patients did not receive tracheostomy [9,11]. In addition, a previous study showed that tracheostomy was not associated with the increased weaning rate of PMV patients in the RCC [12].

Pressure support ventilation (PSV) SBT and T-piece SBT have similar rates of successful weaning, mortality rate, and reintubation rate in adult patients who received mechanical ventilation [13]. In addition, a 30 min PSV SBT has been shown to be associated with a similar reintubation rate but a higher successful extubation rate than a 2 h T-piece SBT in patients who received mechanical ventilation for more than 24 h because more patients in the 30 min PSV group passed the SBT and were thus extubated [14]. This finding supported the use of a shorter, less demanding SBT in patients with simple weaning. Although PSV was associated with a higher weaning rate in patients with simple weaning, unassisted breathing through a T-piece was the most used SBT in endotracheal intubated PMV patients [3,15]. The preference to use T-piece SBT in PMV patients was also supported by the evidence that T-piece SBT was associated with shorter weaning duration in prolonged-weaning patients [16,17].

Unlike tracheostomized PMV patients, endotracheal intubated PMV patients need an additional step to complete the weaning process, which is to remove the endotracheal tube. However, a successful SBT does not guarantee the success of extubation [18]. Older age, longer duration of mechanical ventilation, and the presence of underlying cardiac or respiratory disease were associated with a higher risk of extubation failure [19,20]. These findings suggested that endotracheal intubated PMV patients had a higher risk of extubation failure. Extubation failure is a major complication in PMV patients and is associated with longer ventilator days, longer ICU and hospital stays, and increased hospital mortality [15,21,22].

A shorter and less demanding SBT has been shown to be sufficient to predict the extubation outcome in mechanically ventilated patients in the general ICU [14,23]. However, this approach might not necessarily have similar results in patients who received prolonged mechanical ventilation. A previous study showed that 2 h SBT had low performance in predicting the weaning possibility in PMV patients [24]. The weaning process in PMV patients usually involves a gradual increase in the duration of SBT [2,3,25,26]. Gradual increases in the duration of SBT for up to 12 or 24 h have been proposed in tracheostomized PMV patients and were shown to be useful to differentiate those patients who can be successfully weaned from mechanical ventilation from those who were not weaned [27,28,29]. However, these studies were done in tracheostomized PMV patients, and the results found in these studies might not be extrapolated to endotracheal intubated PMV patients directly. Elderly patients have been shown to be associated with a higher risk of extubation failure [22]. A previous study showed that critically ill elderly patients who can pass 8 h T-piece SBT had lower extubation failure rate than those who passed 2 h T-piece SBT [30]. These findings suggested that T-piece SBT might need to be prolonged in PMV patients because of the higher risk of extubation failure. Nonetheless, the optimal duration of SBT in PMV patients remains uncertain. In this study, we compared the extubation, weaning, and survival outcomes in PMV patients who were extubated after they passed a 12 h T-piece SBT or 24 h T-piece SBT.

## 2. Materials and Methods

### 2.1. Data Collection

This study was approved by the Chang Gung Medical Foundation Institutional Review Board (approval: 201900650B0). The indications for RCC admission were mechanically ventilated for at least 21 days, stable hemodynamic status, stable oxygenation status (FiO_2_ ≤ 40%, and positive end-expiratory pressure ≤ 10 cm H_2_O), no acute hepatic failure or unstable acute renal failure, and not preparing for surgical intervention within the ensuing 2 weeks. Medical records of all PMV patients who were transferred to the RCC in Chiayi Chang Gung Memorial Hospital between May 2009 and September 2014 were reviewed. Patients who received endotracheal intubation and were extubated following a successful SBT were included in this study. The demographics of patients, reason for mechanical ventilation, duration of ICU and RCC stays, number of ventilation days, and Charlson Comorbidity Index (CCI) were recorded. The Acute Physiology and Chronic Health Evaluation II (APACHE II) scores, the highest Glasgow Coma Scale (GCS) scores, and basic laboratory data were collected upon arrival at the RCC. The weaning indices, including respiratory rate, tidal volume, minute ventilation, maximal inspiratory pressure, and rapid shallow breathing index, were measured within 24 h of RCC admission.

### 2.2. The Weaning Process in RCC

The weaning process in the RCC included identification and treatment of the indication of mechanical ventilation and reversible etiologies of difficult weaning, an effort to assess the nutrition status and to maintain adequate nutrition support by an on-site dietitian, and implementation of pulmonary rehabilitation. The options of SBT for endotracheal intubation patients in the RCC include a T-piece, CPAP, and pressure support ventilation (PSV). The weaning program was conducted by gradual reduction of ventilator support followed by stepwise increases in the duration of SBT to 12 or 24 h according to the pre-established RCC weaning protocol. The choice of the method and the duration of SBT were determined according to the preference of the physician. During the study period, the majority of patients were extubated after they passed 12 h T-piece SBT or 24 h T-piece SBT. This result provided the opportunity to compare the extubation and weaning outcomes between 12 h T-piece SBT and 24 h T-piece SBT. There were no differences in these weaning strategies between the 12 h T-piece SBT group and the 24 h T-piece SBT. After a successful SBT, endotracheal intubated patients were connected to a mechanical ventilator for rest before they were extubated, as recommended by a previous study [31].

### 2.3. Definition of Outcomes

“Weaning” was defined as patients who were independent of mechanical ventilation for 5 consecutive days according to the PMV weaning criteria of the National Health Insurance in Taiwan. Patients successfully weaned from mechanical ventilation in the RCC were transferred to the general ward for further rehabilitation and treatment for their medical condition. PMV patients who were not weaned from mechanical ventilation within 42 days in the RCC were transferred to the Respiratory Care Ward (RCW) for further long-term care and ventilator support. Extubation failure was defined as the inability to sustain spontaneous breathing that led to the need for either invasive or non-invasive mechanical ventilation within 5 days after extubation.

### 2.4. Statistical Analysis

Categorical data were expressed as numbers and percentages and were compared using Chi-square/Fisher’s exact tests; continuous data were expressed as mean ± standard deviation and compared by *t*-tests. Kaplan–Meier survival plots and Cox proportional hazard models were used to evaluate the survival risk between groups. Data were analyzed using SPSS 22.0 statistical software (IBM Inc., Armonk, NY, USA). To assess whether the number of patients included in the study is sufficient to support a definitive conclusion, we implemented the sample size calculation method in R, setting HR = 1, testing margin = 0.7, overall probability of the event = 0.26, proportions of the sample size allotted to the two groups = 0.51, type I error = 0.05, and type II error = 0.20. The calculated sample size was 350—close to our actual sample size of this study (n = 354).

## 3. Results

### 3.1. Patient Demographics

**Figure 1:**
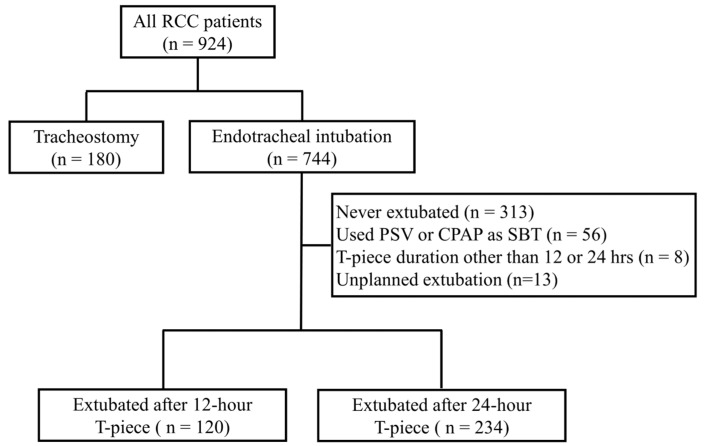
Process of patient selection. RCC—respiratory care center; PSV—pressure support ventilation; CPAP—continuous positive airway pressure; SBT—spontaneous breathing trial.

A total of 924 patients were admitted to the RCC during the study period. Excluded from this study were patients tracheostomized before weaning attempt (n = 180), not extubated in the RCC (n = 313), extubated after passing a continuous positive airway pressure (CPAP) or pressure support ventilation (PSV) SBT (n = 56), extubated after passing a T-piece duration other than 12 or 24 h (n = 8), and those who had unplanned extubated (n = 13) (Figure 1). A total of 354 patients were included for further analysis. Among these 354 patients, 120 of them were extubated after passing a 12 h T-piece SBT. The other 234 patients were extubated after passing a 24 h T-piece SBT. These two groups of patients had no significant difference in age, gender, number of ventilator days, etiologies of mechanical ventilation, or weaning indices upon RCC arrival (Table 1). Patients in the 24 h T-piece SBT group had higher APACHE II scores and lower GCS scores upon RCC arrival than patients in the 12 h T-piece SBT group. Although more patients in the 24 h T-piece SBT group had mild liver disease, there was no significant difference in the CCI between these two groups of patients (Table 2). Before extubation, there was no difference in the number of ventilator days between these two groups of patients (Table 3). Patients in the 24 h T-piece SBT group had higher PImax than patients in the 12 h T-piece SBT group before extubation. However, there was no difference in other weaning indices between these two groups of patients. Although patients in the 24 h T-piece SBT group had higher arterial blood pH values than those in the 12 h T-piece SBT group, these two groups had no differences in all the other parameters of arterial blood gas analysis after SBT.

### 3.2. RCC Extubation and Weaning Outcome

**Figure 2:**
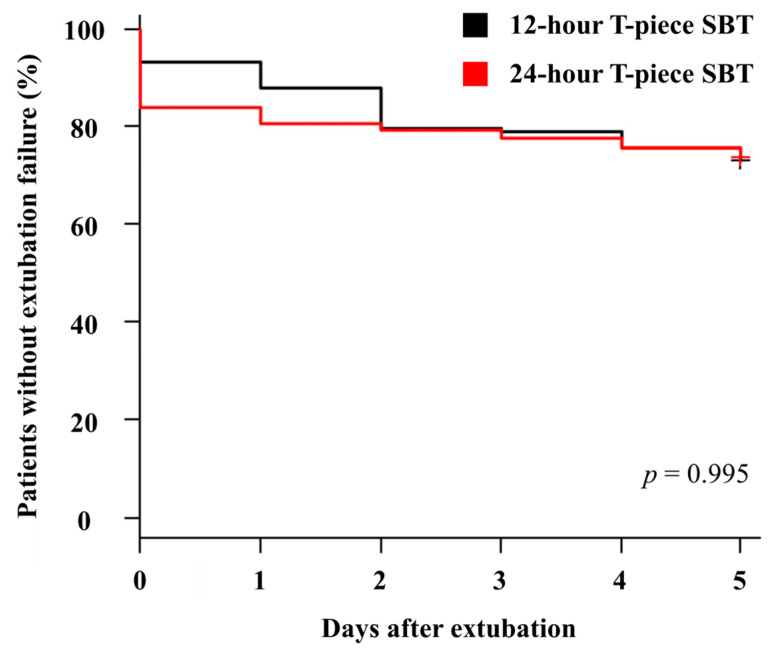
Kaplan–Meier survival curves for extubation failure in patients who were extubated after they passed 12 h T-piece spontaneous breathing trial (SBT, black) or 24 h T-piece SBT (red). SBT—Spontaneous breathing trial.

After extubation, 32 patients (26.7%) in the 12 h T-piece SBT group and 61 patients (26.1%) in the 24 h T-piece SBT group had extubation failure (Table 4). There was no difference in ventilatory management of extubation failure between these two groups of patients. Kaplan–Meier survival analysis showed no significant difference in the cumulative probability of extubation failure between these two groups of patients within 5 days after extubation (Figure 2). There was no difference in RCC mortality between patients in the 12 h T-piece SBT group (n = 7, 5.8%) and patients in the 24 h T-piece SBT group (n = 15, 6.4%). Among patients who had extubation failure, 14 patients in the 12 h T-piece SBT group and 27 patients in the 24 h T-piece SBT group were successfully weaned later. The RCC weaning rates were similar between the 12 h T-piece SBT group (n = 102, 85%) and the 24 h T-piece SBT group (n = 200, 85.5%). In addition, there was no significant difference in the length of RCC stay between these two groups of patients.

### 3.3. Hospital Outcome

After successfully weaning in the RCC and transferring to the general ward, some of the patients in both groups had respiratory failure and thus reinstitution of mechanical ventilation before hospital discharge. The final hospital weaning rate in the 12 h T-piece SBT group (n = 84, 70%) was not different from that in the 24 h T-piece SBT group (n = 156, 66.7%). In addition, these two groups of patients had no difference in the hospital mortality rate.

### 3.4. Extubation Outcomes in Elderly, Myocardial Infarction, Heart Failure, or Cerebrovascular Disease Patients

In this study, 217 patients had ages older than 70 years. Among them, 67 patients were extubated after passing 12 h T-piece SBT and 150 patients were extubated after passing 24 h T-piece SBT. In patients younger than 70 years, 53 patients were extubated after passing 12 h T-piece SBT, and 84 patients were extubated after passing 24 h T-piece SBT. The extubation failure rate was similar between the 12 h T-piece SBT and 24 h T-piece SBT in patients older than 70 (23.9% vs. 24.7%, *p* = 0.901) and in patients younger than 70 (30.2% vs. 28.6%, *p* = 0.839) (Table 5). Our data showed that 12 h T-piece SBT and 24-T-piece SBT had similar extubation outcomes in PMV patients older or younger than 70. In addition, there was no difference in the extubating failure rate between 12 h T-piece SBT and 24 h T-piece SBT in patients with cerebrovascular disease. On the other hand, 24 h T-piece SBT was associated with a lower extubation failure rate than 12 h T-piece SBT in patients with myocardial infarction or heart failure. These findings suggested that although 24 h T-piece SBT was not associated with a lower extubation failure rate in all enrolled PMV patients in this study, it was associated with a lower extubation failure rate in PMV patients with myocardial infarction or heart failure.

## 4. Discussion

A common SBT for PMV patients is usually with an unassisted T-piece with a progressive increase in the duration of SBT beyond the 30 min or 120 min limit in the ICU [3]. A previous study showed that the 8 h T-piece SBT was associated with a lower extubation failure rate than the 2 h T-piece SBT in critically ill and older patients receiving mechanical ventilation [25]. This finding suggested that SBT might need to be prolonged in patients at higher risk of extubation failure. However, the optimal SBT duration remained uncertain. In this study, we showed that the extubation outcomes, weaning outcomes, and survival outcomes were similar in PMV patients who were extubated after the 12 h T-piece SBT and 24 h T-piece SBT. This finding was supported by two pieces of evidence. First, the extubation failure rates were similar after PMV patients passed a 12 h or 24 h T-piece SBT. Second, these two groups of patients had no differences in the RCC weaning rate, RCC mortality rate, hospital weaning rate, and hospital mortality rate.

Patients who received prolonged mechanical ventilation were known to have poor prognosis [7,32]. A meta-analysis of 29 high-quality PMV studies showed a pooled mortality rate of 29% at hospital discharge and a pooled mortality rate of 62% at 1 year [8]. Only 50% of patients were successfully liberated from mechanical ventilation in 30 high-quality PMV studies [8]. Successful weaning from prolonged mechanical ventilation improved survival and reduced the utilization of healthcare resources [33,34]. The weaning process for endotracheal intubated PMV patients consists of two steps: gradual reduction in the level of ventilatory support and removal of the endotracheal tube. A substantial proportion of mechanically ventilated patients passed the spontaneous breathing trial but failed the extubation step of the weaning process [35,36,37]. The prolonged duration of mechanical ventilation was associated with an increased risk of extubation failure [22,38]. Extubation failure was associated with significantly longer ICU stays, longer hospital stays, and higher mortality rates [22]. It is crucial to reduce the risk of extubation failure in PMV patients.

Older age, longer duration of mechanical ventilation, and the presence of underlying cardiac or respiratory disease have been shown to be associated with a higher risk of extubation failure in patients who received mechanical ventilation [19,20,22]. However, a previous study showed that the extubation rate was lower in the 8 h T-piece SBT than in the 2 h T-piece SBT in elderly patients with critical illness [30]. Our result showed that an increase in the duration of the T-piece SBT from 12 h to 24 h was not associated with a lower extubation failure rate. In this study, we compared the extubation outcomes between 12 h T-piece SBT and 24 h T-piece SBT in patients with myocardial infarction or heart failure. We found that PMV patients with myocardial infarction or heart failure had lower extubation failure rates after passing 24 h T-piece SBT than 12 h T-piece SBT. Cardiac dysfunction is one of the important factors associated with extubation failure [39]. This finding suggested that SBT might need to be prolonged in this group of patients.

Longer RCC stays, elevated blood urea nitrogen (BUN) levels, and lower modified GCS scores, serum albumin, and maximal inspiratory pressure (PImax) levels have been shown to be predictors of unsuccessful weaning from prolonged mechanical ventilation in RCC [9]. The two groups in this study did not differ significantly in length of RCC stay or serum BUN or albumin levels. The APACHE II score upon RCC arrival was higher in the 24 h T-piece SBT group than in the 12 h T-piece SBT group. A higher APACHE II score had been shown to be associated with unsuccessful weaning from mechanical ventilation in all PMV patients admitted to RCC [9]. However, weaning predictors may not be good predictors of extubation failure [37]. In addition, the included patients in this study were PMV patients who were extubated after they passed the T-piece SBT and were medically stable. The APACHE II score upon RCC arrival may not represent the disease severity of patients at the time when they were extubated.

Respiratory rate, tidal volume, and RSBI have been shown to be predictors of extubation failure in mechanically ventilated patients who were extubated after they passed the T-piece SBT [37]. In this study, the two groups of patients had no difference in respiratory rate, tidal volume, minute ventilation, or RBSI. The PImax before extubation was higher in the 24 h T-piece SBT group than in the 12 h T-piece SBT group. Higher PImax was shown to be associated with successful weaning from mechanical ventilation in PMV patients admitted to the RCC [9]. Despite the favorable PImax in the 24 h T-piece SBT group, the two groups had no difference in the extubation failure rate, the RCC length of stay, the RCC weaning rate, or the hospital weaning rate. Our finding suggested that, although PImax is a predictor of successful weaning from mechanical ventilation in PMV patients admitted to the RCC, it might not be a good predictor of extubation failure in PMV patients who were extubated after they passed the T-piece SBT.

Although the arterial blood pH after SBT was higher in the 24 h T-piece SBT group than in the 12 h T-piece SBT group, a previous study showed no difference in arterial blood pH after T-piece SBT between extubation success patients and extubation failure patients [37]. This finding suggested that arterial pH after T-piece SBT might not be a predictor of extubation failure. On the other hand, arterial blood PaO_2_ and SaO_2_ after T-piece SBT were lower in extubation failure patients than in extubation success patients [37]. These findings suggested that arterial blood PaO_2_ and SaO_2_ after T-piece SBT might be predictors of extubation failure. The two groups of patients in this study had no difference in the arterial blood PaO_2_ and SaO_2_. The results of arterial blood gas analysis and extubation outcomes in this study were consistent with those of the previous report [37].

The focus of this study is to investigate whether prolonging T-piece SBT beyond 12 h might reduce the risk of extubation failure in PMV patients. PMV patients are a group of patients with heterogenous causes of critical illness, etiologies of mechanical ventilation, and underlying comorbidities. Previous studies showed that PMV patients had a higher risk of extubation failure than patients who received mechanical ventilation for a shorter period [19,20]. A longer and more demanding SBT might be needed in PMV patients to reduce the risk of extubation failure [27,28,29,30]. In this study, we found that prolonging SBT beyond 12 h might not reduce the risk of extubation failure in PMV patients. However, we also found that extubation after passing 24 h T-piece SBT was associated with a lower risk of extubation failure than extubation after passing 12 h T-piece SBT in PMV patients with myocardial infarction or heart failure. These findings suggested that a single SBT strategy might not be sufficient to predict extubation outcomes for all PMV patients. In addition, the possible benefit of prolonged T-piece SBT in reducing the risk of extubation failure in PMV patients should be weighed against the disadvantage of delayed extubation. Gradually, an increase in the duration of SBT during the weaning process in PMV patients inevitably delayed the timing of extubation. However, extubation failure is associated with increased morbidity and mortality in PMV patients [15,21,22]. Delayed extubation has been shown to be associated with an increased risk of ventilator-associated pneumonia, longer ICU stay, laryngotracheal injury, extubation failure, and mortality [22,40,41,42,43]. Further prospective randomized studies will be needed to investigate the patient characteristics that predict a higher risk of extubation failure and to determine the optimal SBT strategy for these PMV patients.

There were limitations in this study. First, this is a retrospective study performed in a single center. The choice of the duration of SBT was determined according to the RCC weaning protocol and the preference of the physician. Because of the retrospective nature of this study, the choice between a 12 h T-piece SBT and a 24 h T-piece SBT was not randomized, and the possibility of indication bias cannot be completely excluded. The findings found in this study will need to be validated by further prospective multicenter randomized studies. Second, patients who failed SBT or those who were extubated after a successful CPAP SBT were not included in this study. Thus, the findings in patients who received the T-piece SBT may not be extrapolated to patients who received other SBT methods, such as PSV or CPAP. Third, the impact of pulmonary rehabilitation on the weaning outcome was not assessed in this study. Implementing pulmonary rehabilitation reduced ICU-acquired weakness in patients who received mechanical ventilation [44]. Breathing training and limb muscle training have been shown to be associated with higher survival rates in PMV patients [45]. In addition, inspiratory muscle strength training improved maximal inspiratory pressure in patients who received mechanical ventilation and improved weaning outcomes in failure-to-wean patients [46,47]. In this study, pulmonary rehabilitation, including inspiratory muscle strength training and upper and lower-extremity exercises in bed, was aggressively integrated into the weaning program after the PMV patients were admitted to the RCC, and there was no difference between the two groups of patients regarding the prescription of rehabilitation modalities. However, the retrospective nature of this study limited the feasibility of investigating the impact of pulmonary rehabilitation on the weaning and survival outcomes of PMV patients because the duration and sessions of rehabilitation increased along with the duration of RCC stay in the enrolled patients.

## 5. Conclusions

In this study, we found that the extubation, weaning, and survival outcomes were similar in the PMV patients who were extubated after the successful 12 h T-piece SBT or the 24 h T-piece SBT. However, subgroup analysis showed that the 24 h T-piece SBT might be associated with a lower extubation failure rate in PMV patients with myocardial infarction or heart failure.

## Figures and Tables

**Table 1:** Patient characteristics upon Respiratory Care Center arrival.

	12 h T-Piece SBT	24 h T-Piece SBT	
Characteristics	(n = 120)	(n = 234)	*p*
Age (years)	69.68 ± 14.73	72.46 ± 14.24	0.085
Gender			
Male	71 (59.2%)	129 (55.1%)	0.468
Female	49 (40.8%)	105 (44.9%)	
Ventilator days	22.23 ± 2.84	22.76 ± 5.67	0.295
Etiology of MV			
Acute lung disease	32 (26.7%)	73 (31.2%)	0.586
Chronic lung disease	3 (2.5%)	5 (2.1%)	
Cardiac disease	8 (6.7)	23 (9.8%)	
Neurologic disease	43 (35.9%)	80 (34.2%)	
Post-operation	8 (6.7%)	18 (7.7%)	
Miscellaneous	26 (21.7%)	35 (15%)	
APACHE II score	25.07 ± 4.07	26.37 ± 4.68	0.01
Glasgow Coma Scale	8.99 ± 1.99	8.5 ± 2.46	0.046
Eye	3.19 ± 1.20	3.04 ± 1.32	0.286
Motor	4.77 ± 1.10	4.46 ± 1.40	0.038
Charlson comorbidity index	4.71 ± 2.61	5.10 ± 2.94	0.172
BUN	35.58 ± 37.74	37.34 ± 34.86	0.663
Cr	1.39 ± 1.80	1.53 ± 2.96	0.625
Albumin	2.17 ± 0.50	2.39 ± 1.46	0.109
Weaning profiles			
RR	27.58 ± 6.9	26.28 ± 6.4	0.08
TV	328.48 ± 116.32	326.92 ± 105.38	0.899
MV	8.78 ± 3.08	10.13 ± 25.87	0.571
PImax	−23.78 ± 1.00	−24.90 ± 12.14	0.388
RSBI	98.35 ± 55.01	90.18 ± 44.76	0.135

Data indicates value ± standard deviation or number (%) of patients. Abbreviations: MV—mechanical ventilation; BUN—blood urea nitrogen; Cr—creatinine; RR—respiratory rate; TV—tidal volume; PImax—maximal inspiratory pressure; RSBI—rapid shallow breathing index.

**Table 2:** Comorbidities of included patients upon Respiratory Care Center arrival.

		12 h T-Piece SBT	24 h T-Piece SBT	*p*
		(n = 120)	(n = 234)	
Myocardial infarction		12 (10%)	30 (12.8%)	0.437
Heart failure		21 (17.5%)	47 (20.1%)	0.559
Peripheral vascular disease		23 (19.2%)	34 (14.5%)	0.261
Cerebrovascular disease		71 (59.2%)	150 (64.1%)	0.364
COPD		5 (4.2%)	11 (4.7%)	0.819
Connective tissue disease		2 (1.7%)	2 (0.9%)	0.607
Peptic ulcer disease		46 (38.3%)	69 (29.5%)	0.093
Liver disease				
	Mild	34 (28.3%)	39 (16.7%)	0.01
	Moderate-severe	3 (2.5%)	12 (5.1%)	0.403
DM				
	Without target organ damage	33 (27.5%)	68 (29.1%)	0.758
	With target organ damage	26 (21.7%)	44 (18.8%)	0.522
Dementia		17 (14.2%)	37 (15.8%)	0.684
Hemiplegia		55 (45.8%)	129 (55.1%)	0.098
Solid tumor		16 (13.3%)	25 (10.7%)	0.461
Lymphoma		0 (0%)	2 (0.9%)	0.551
Leukemia		0 (0%)	0 (0%)	-
Moderate-Severe renal disease		25 (20.8%)	39 (16.7%)	0.335
Metastatic solid cancer		6 (5%)	7 (3%)	0.342
AIDS		0 (0%)	0 (0%)	-

Data indicate the number (%) of patients. Abbreviations: COPD—chronic obstructive pulmonary disease; DM—diabetes mellitus; AIDS—acquired immunodeficiency syndrome.

**Table 3:** Respiratory mechanics before extubation and arterial blood gas analysis after spontaneous breathing trial.

	12 h T-Piece SBT	24 h T-Piece SBT	
Characteristics	(n = 120)	(n = 234)	*p*
Ventilator days before extubation	39.48 ± 10.11	38.47 ± 9.87	0.365
Weaning profiles			
RR	24.87 ± 5.73	24.43 ± 6.06	0.511
TV	364.37 ± 108.12	363.58 ± 101.91	0.946
MV	8.96 ± 2.75	8.56 ± 2.52	0.178
PImax	−25.76 ± 9.12	−28.03 ± 10.13	0.04
RSBI	80.74 ± 72.04	74.28 ± 35.65	0.261
Arterial blood gas analysis after SBT			
pH	7.45 ± 0.05	7.48 ± 0.04	<0.001
PCO_2_	41.14 ± 8.33	40.36 ± 6.45	0.434
PO_2_	127.92 ± 42.78	125.77 ± 46.96	0.71
HCO_3_	28.99 ± 5.43	30.09 ± 4.48	0.068
SatO_2_	0.98 ± 0.02	0.98 ± 0.06	0.568
FiO_2_	0.395 ± 0.101	0.385 ± 0.099	0.365

Values indicate mean ± standard deviation or number (%) of patients. Abbreviations: SBT—spontaneous breathing trial; RR—respiratory rate; TV—tidal volume; MV—mechanical ventilation; PImax—maximal inspiratory pressure; RSBI—rapid shallow breathing index.

**Table 4:** Extubation, weaning, and survival outcomes of included prolonged mechanical ventilation patients.

	12 h T-Piece SBT	24 h T-Piece SBT	
Outcome	(n = 120)	(n = 234)	*p*
Extubation failure	32 (26.7%)	61 (26.1%)	0.904
Ventilatory management for extubation failure			
IMV	7 (5.8%)	19 (8.1%)	0.311
NIV	16 (13.3%)	32 (13.7%)	
NIV + IMV	6 (5%)	4 (1.7%)	
DNI	3 (2.5%)	6 (2.6%)	
RCC stay (days)	25.35 ± 10.31	24.17 ± 11.07	0.331
RCC discharge status			
Weaned	102 (85%)	200 (85.5%)	0.929
Not weaned	11 (9.2%)	19 (8.1%)	
Mortality	7 (5.8%)	15 (6.4%)	
Hospital stay (days)	79.83 ± 69	71.20 ± 39.59	0.136
Hospital discharge status			
Weaned	84 (70%)	156 (66.7%)	0.821
Not weaned	13 (10.8%)	27 (11.5%)	
Mortality	23 (19.2%)	51 (21.8%)	

Values indicate mean ± standard deviation or number (%) of patients. Abbreviations: IMV—invasive mechanical ventilation; NIV—non-invasive mechanical ventilation; DNI—do not intubate; RCC—respiratory care center.

**Table 5:** Extubation failure rate in elderly, myocardial infarction, heart failure, or cerebrovascular disease patients.

		Extubation Success	Extubation Failure	*p*
Age > 70				
	12 h T-piece SBT	51 (76.1%)	16 (23.9%)	0.901
	24 h T-piece SBT	113 (75.3%)	37 (24.7%)	
Age ≤ 70				
	12 h T-piece SBT	37 (69.8%)	16 (30.2%)	0.839
	24 h T-piece SBT	60 (71.4%)	24 (28.6%)	
Myocardial infarction				
	12 h T-piece SBT	4 (33.3%)	8 (66.7%)	0.008
	24 h T-piece SBT	23 (76.7%)	7 (23.3%)	
Heart failure				
	12 h T-piece SBT	11 (52.4%)	10 (47.6%)	0.046
	24 h T-piece SBT	36 (76.6%)	11 (23.4%)	
Cerebrovascular disease				
	12 h T-piece SBT	53 (74.6%)	18 (25.4%)	0.918
	24 h T-piece SBT	111 (74%)	39 (26%)	

Value indicates the number (%) of patients.

## Data Availability

The authors declare that the data for this research are available from the correspondence authors upon reasonable request.
